# Napier grass (*Pennisetum purpureum* Schum) management strategies for dairy and meat production in the tropics and subtropics: yield and nutritive value

**DOI:** 10.3389/fpls.2023.1269976

**Published:** 2023-11-14

**Authors:** M. Rafiq Islam, Sergio C. Garcia, Nathu R. Sarker, Md. Ashraful Islam, Cameron E. F. Clark

**Affiliations:** ^1^ Dairy Science Group, School of Life and Environmental Sciences, Faculty of Science, The University of Sydney, Camden, NSW, Australia; ^2^ Livestock Production and Welfare Group, School of Life and Environmental Sciences, Faculty of Science, The University of Sydney, Camden, NSW, Australia; ^3^ Sydney Institute of Agriculture, Faculty of Science, The University of Sydney, Camden, NSW, Australia; ^4^ Krishi Gobeshona Foundation, Bangladesh Agricultural Research Council Complex, Dhaka, Bangladesh; ^5^ Department of Dairy Science, Faculty of Animal Science and Veterinary Medicine, Patuakhali Science and Technology University, Barishal, Bangladesh

**Keywords:** smallholder farmers, elephant grass, sowing density, harvesting frequency, food security, best management practice

## Abstract

Napier grass (*Pennisetum purpureum* Schumach) comprises up to 80% of the cattle diet in many tropical and subtropical regions and is used primarily by smallholder farmers. Despite the grass’s high yield, resulting animal productivity from this grass is low. One of the key reasons for the low animal productivity of Napier grass is its low nutritive value under current management. Taken together, previous work has shown the current yield, crude protein (CP), and metabolisable energy (ME) of Napier grass to be 26 t dry matter (DM)/ha/year, 96 g/kg DM, and 8.7 MJ/kg DM, respectively, ranging from 2 to 86 t DM/ha/year, 9 to 257 g CP/kg DM, and 5.9 to 10.8 MJ ME/kg DM, respectively, suggesting an opportunity for significant improvement on both yield and nutritive value of this grass. The DM yield and nutritive value of this grass are inversely related, indicating a trade-off between yield and quality; however, this trade-off could be minimised by increasing sowing density and harvesting frequency. Available literature shows that this simple management strategy of increasing sowing density (50 cm × 40 cm) and harvesting frequency (11–12 harvests/year) provides 71 t DM/ha with 135 g/kg DM CP and 10.8 MJ ME/kg DM. This quality of Napier grass has the potential to increase both milk and meat production substantially in the tropics and subtropics, and the farmers will likely find this simple management acceptable due to the high yield obtained through this management. However, there is a paucity of work in this field. Therefore, management strategies to improve the nutritive value of Napier grass are required to increase milk and meat production in the tropics and subtropics and in doing so improve the food security of more than half of the global population living in these regions.

## 1 Introduction

Napier grass (*Pennisetum purpureum* Schumach) is the key diet of many elephants, and as such, is commonly known as elephant grass (Heuzé et al., 2020) but it may also be named as elephant grass due to its robust growth as opposed to other grass species. The taxon *Cenchrus purpureus* (Schumach) was also proposed in 2010 as a replacement for *P. purpureum* Schumach (Chemisquy et al., 2010). Whilst native to Sub-Saharan tropical Africa, the robust growth of this grass makes it one of the most popular and important forages for smallholder livestock farmers in the tropics and subtropics (Muktar et al., 2022). As a result, this grass has been introduced and naturalised in more than 100 tropical and subtropical countries throughout the world, particularly in Africa, Asia, and Latin America (Cook et al., 2005; Clayton et al., 2013), representing over 50% of the world’s population (Almeida, 2018). As such, Napier grass in the tropics and subtropics is as popular as perennial ryegrass (*Lolium perenne*) in the temperate region for animal production. The tropics is a vast area encompassing most of Central and South America, the Caribbean, Africa, India, Southeast Asia, Northern Australia, and most of the Pacific Islands. The subtropics encompass the Southern USA, the Mediterranean, Northern India, and China to the North and South Africa, Australia, and Southern Brazil to the South, where Napier grass or similar species are cultivated widely.

Napier grass is a world record holder grass for yield at 86 t dry matter (DM)/ha (Vicente-Chandler et al., 1959). This high-yielding characteristic makes it highly popular with smallholder farmers in tropical and subtropical farmers. Therefore, up to 80% of the forage ingested by cows in many tropical and subtropical countries is Napier grass (Kabirizi et al., 2015), and focus of farmers in these areas are high biomass yield (40%–59%; associated with plant height) and rapid re-growth (10%–26%). Smallholders typically harvest Napier grass when it reaches between 2 and 3 m in height (Zhang et al., 2010; Rengsirikul et al., 2013) in order to maximise yield from their small and fragmented areas of land. Under such growth priority-based current management, this grass currently provides low crude protein (CP; 70–100 g/kg DM) and metabolisable energy (ME; ~8 MJ/kg DM) for ruminant production (Cook et al., 2005), as there is a trade-off between growth and quality. Consequently, despite high yield, the low CP and ME of this grass obtained under current management cannot meet the protein and energy requirements of beef and dairy animals with consequences for both productivity (Muia et al., 2001a; Muia et al., 2001b). In addition, animals offered with such low-quality Napier grass usually require high amounts of grain-based concentrate to support milk and meat production. As such, due to the requirement of high-cost feeds (suitable for a monogastric production system), milk and meat production costs in Napier grass-growing countries are high (Roy et al., 2017) and often similar to or higher than the costs in international markets. Consequently, Napier grass-growing tropical and subtropical countries including Bangladesh are the main importers of beef and dairy products. As a result, there is insufficient consumption of animal-sourced food, and 815–821 million people in these regions suffer from hunger and remain undernourished (Islam et al., 2021). In contrast, ryegrass (*L. perenne*) offered to cattle in temperate countries usually contains high CP (240 g/kg DM) and ME (11 MJ/kg DM), which alone (i.e., without supplementation) can support up to 22 L of milk (Fulkerson, 2007). With such nutrient content in the grass, temperate countries are able to produce milk and meat abundantly at a low cost and are exporters of milk and meat. Fulkerson et al. (2010) reported that Kikuyu grass (*Pennisetum clandestinum* ex chiov), a C_4_ *Pennisetum* species similar to Napier grass, contains ~20 g CP/kg DM and ~10 MJ/kg DM at 16 days of harvest interval (HI; and 4.5 leaf stage), which could be taken as an ideal quality of Napier grass. The range of CP (45–243 g/kg DM) and ME (6.68–9.58 MJ/kg DM) reported by Habte et al. (2022) suggests that it is also possible to manage Napier grass in the tropics as high quality as ryegrass in the temperate zone, or similar to the Kikuyu grass (Fulkerson et al., 2010). Therefore, one option to reduce production costs and to increase the productivity of both milk and meat is to markedly improve the nutritive value of Napier grass to the level of ryegrass or Kikuyu grass through management such as inputs (e.g., fertiliser and water), variety, and agronomic management if tropical countries target to produce milk and meat at an internationally competitive rate and to become self-sufficient in both products. Islam et al. (2021) in a review reported that there is an immense opportunity to at least double the levels of ruminant food production through simple changes in Napier grass management from the same land area to improve food security and reduce malnutrition across vast tropical areas. Under this context, investigation and development of management strategies are needed to improve both yield and quality simultaneously to derive a feed that optimises animal production and health whilst minimising overall feed costs.

The main aim of this review is to identify and investigate different management factors to optimise Napier grass yield and nutritive value for animal production in subtropical/tropical regions. This review will cover yield and nutritive value from Napier grass under current management, identify gaps, and investigate ways to develop best management practice (BMP) to improve both yield and quality of this grass so that millions of smallholders living in hundreds of countries in the tropics and subtropics find ways to increase milk and meat from their animals.

## 2 Morphology and habitat of Napier grass

Napier is a C_4_ perennial grass in the Poaceae family. It can grow up to 7.5 m in height, and its extensive root system can penetrate up to 4.5 m, which makes it a highly drought-tolerant grass (Cook et al., 2005) and potentially important in carbon sequestration (Yang et al., 2019). It has a thick stem near the base (3-cm diameter) with long (up to 120 cm) and wide leaf blades (up to 5 cm). It has vigorous tillering, large leaf area, high solar radiation interception and radiation use efficiency, tall canopy (Kubota et al., 1994), and high photosynthetic rate and can maintain radiation use efficiency for a long time as compared to other C_4_ plants (Ito and Inaga, 1988). The average tiller per plant is 35 (Amin et al., 2016) to 100, depending on season and variety (Macoon et al., 2002). Napier’s leaf-to-stem ratio (L:S) is 0.57–1.63 (Halim et al., 2013), and dwarf varieties contain more leaves compared to stems. It grows well in full sunlight (Anderson et al., 2008) but can also grow under partial shade (Francis, 2004). Napier grass has all the fundamental factors for high productivity such as vigorous tillering, large leaf area, high photosynthetic rate, and tall canopy and has greater growth potential than maize (Ito and Inaga, 1988; Matsuda et al., 1991).

The common name “Napier grass” comprises approximately 140 species; over 300 accessions are available in various gene banks around the world (Negawo et al., 2017). It grows in a wide range of soil and climatic conditions ranging from low fertility acid soils to slightly alkaline soils (Hanna et al., 2004). However, it grows well on rich, deep, and well-drained loamy soils under a pH range of 4.5 to 8.2 (Duke, 1983; Cook et al., 2005). It spreads by rhizomes, and farmers propagate it vegetatively mainly by stem cuttings, as this grass cannot produce many effective seeds for propagation (Singh et al., 2013). Napier grass grows from sea level to 2,000 m of altitude and in rainfall ranging from 200 to 4,000 mm but grows best between 750 and 2,500 mm of rainfall per annum (Negawo et al., 2017). However, it does not tolerate prolonged waterlogging conditions lasting for more than 3 days (Nelson, 2005). It thrives in highlands and arid environments of Africa (Kabirizi et al., 2015; Kebede et al., 2017) mainly because of its extensive root system, and the grass grows well in saline conditions (Rahman and Talukder, 2015). The optimum temperature for its growth is 33°C during the day and 28°C during the night (Ferraris et al., 1986) and grows well in temperatures between 25°C and 40°C (Duke, 1983). Napier grass is highly popular with smallholder farmers because of its high yield, fast regrowth, drought tolerance, and suitability for cut-and-carry systems and is easy to establish. Napier grass can supply forage year-round for more than 8 years once established (Sollenburger et al., 1989); thus, it is a low-maintenance grass for smallholder tropical and subtropical farmers.

## 3 Current Napier grass production systems

Napier grass yield varies widely from 2 (Bogdan, 1977) to 86 t DM/ha/year (Vicente-Chandler et al., 1959) with a mean of 28 t DM/ha/year (**Table 1**). More than 50 t DM/ha/year from this grass is reported in many countries around the world (**Table 1**). High yield under experimental plot-level studies was recorded in the USA (78 t DM/ha; Goorahoo et al., 2005), China (74 t DM/ha; Zhang et al., 2010), Thailand (71 t DM/ha; Wijitphan et al., 2009), and Australia (50 t DM/ha; Ferraris, 1980). The maximum yield (86 t DM/ha; Vicente-Chandler et al., 1959) of this grass recorded is ~3 times greater than the maximum recorded yield of Kikuyu grass (30 t DM; 600 kg N/ha; Henzell, 1968) and perennial ryegrass (28 t DM/ha; Neal et al., 2010), which is widely used in the temperate region for successful commercial animal production system. Record high yield for Napier grass usually with non-limiting inputs is not surprising, as Garcia et al. (2014) estimated that the maximum theoretical and potential yields of C_4_ plants based on their highest photosynthesis are 259 and 191 t DM/ha/year, respectively. Yield of Napier grass at smallholder farmer (*n* = 33 farms) level was 57 t DM/ha/year (fresh yield, 314.5 t/ha/year, considering 180 g DM/kg; Roy et al., 2016), which is 66% of the recorded highest yield (86 t DM/ha/year; Vicente-Chandler et al., 1959).

**Table 1:** Available research (1980 to date) on Napier grass management and its yield and nutritive value^1^.

	Average	Minimum	Maximum	*n*
Yield, t dry matter (DM)/ha/year	30.0	2.47	78.1	418
No. of harvest	5.8	1.0	13.0	50
Plant height (cm)	201	50	429	78
Harvest interval (days)	63	14	180	132
Harvest intensity (severity) (cm)	14	5	62	25
Row–row distance (cm)	87	50	100	23
Plant–plant distance (cm)	54	35	100	23
Nitrogen (N, kg/ha)	320	0	2223	79
Phosphorous (P, kg/ha)	102	20	550	32
Potassium (K, kg/ha	212	13	600	27
Irrigation (mm)	722	70	2022	9
Leaf area index (LAI)	7.1	2.5	8.5	6
Leaf:stem ratio	0.8	0.3	2.4	47
Leaf%	47	44	50	2
Dead leaf%	7	7	8	2
Chemical composition (g/kg DM or as stated)				
DM (g/kg)	193	86	380	89
Ash	130	37	250	116
Crude protein (CP)	95	9	257	229
Non-protein N (NPN, g/kg N)	242	242	242	1
Nitrate N (NO_3_–N)	0.5	0.1	0.8	7
Ether extract (EE)	27	12	59	22
Water-soluble carbohydrate (WSC)	97	12	174	25
Non fibre carbohydrate (NFC)	111	76	149	5
Starch	55	39	71	2
Neutral detergent fibre (NDF)	641	479	791	173
Acid detergent fibre (ADF)	388	256	645	137
Lignin	57	20	129	96
Acid detergent insoluble N (ADIN)	7	1	13	2
Cellulose	331	198	473	36
Hemicellulose	262	190	357	39
Silica	57	53	60	4
Total oxalate	25	1	39	15
Insoluble oxalate	7	4	11	9
Soluble oxalate	29	15	34	10
Calcium (Ca)	52.3	0.4	119.4	25
Phosphorous (P)	1.9	0.2	4.4	25
Glucose	62	53	71	8
Gross energy (GE, MJ/kg)	16	15	17	16
Metabolisable energy (ME, MJ/kg DM)	8.6	5.9	10.8	35

^1^Sources: Ferraris, 1980; Brown et al., 1988; Anindo and Potter, 1994; Aroeira et al., 1999; Filho et al., 2000; Huque et al., 2001; Gwayumba et al., 2002; Islam et al., 2003; Aganga et al., 2005; Goorahoo et al., 2005; Chen et al., 2006; Castillo et al., 2010; Das et al., 2010; Bureenok et al., 2012; Danes et al., 2013; Ferreira et al., 2013; Halim et al., 2013; Gomide et al., 2015; Amin et al., 2016; Aswanimiyuni et al., 2018; Garcez et al., 2018; Gusmao et al., 2018; Habte et al., 2022; Ito et al., 1988; Kaitho and Kariuki, 1998; Kariuki et al., 1998; Kariuki et al., 1999; Kozloski et al., 2003; Kozloski et al., 2005; Khairani et al., 2013; Knoll et al., 2013; Kabirizi et al., 2015; Khota et al., 2018; Lounglowan et al., 2014; Manyawu et al., 2003a; Manyawu et al., 2003b; Manyawu et al., 2003c; Magalhães et al., 2010; Muinga et al., 1992; Muinga et al., 1993; Nsahlai et al., 2000; Muia et al., 2001a; Muia et al., 2001b; Muyekho et al., 2015; Parsons et al., 2012; Pieterse and Rethman, 2002; Rahman et al., 2010; Rahman et al., 2013; Rahman et al., 2013; Rahman et al., 2015; Ruiz et al., 1992; Rao et al., 1993; Schank et al., 1993; Sarwar et al., 1999; Rengsirikul et al., 2013; Rahman et al., 2014a; Ramadhan et al., 2015; Roy et al., 2016; Schneider et al., 2018; Sileshi et al., 1996; Shem et al., 2003; Sidhu et al., 2011; Skerman and Riveros, 1990; Tamada et al., 1999; Tessema and Baars, 2004; Tessema, 2008; Vicente-Chandler et al., 1959; Woodard and Prine, 1993; Vieira et al., 1997; Wijitphan et al., 2009; Tessema et al., 2010; Yammeun-art et al., 2017; Yokota et al., 1998; Zhang and Kumal, 2000; Zewdu et al., 2002; Zhang et al., 2010.

Despite high yield, the nutritional quality of Napier grass is low, which cannot often maintain the productivity of livestock. It contains low CP (95 g/kg DM) and ME (8.6 MJ/kg DM) but high acid detergent fibre (ADF; 388 g/kg DM) and neutral detergent fibre (NDF; 641 g/kg DM) (**Table 1**; Cook et al., 2005). However, there is a wide range of variation in nutritive value; for instance, CP (9–257 g/kg DM), ADF (256–645 g/kg DM), NDF (479–791 g/kg DM), and ME (5.9–10.8 MJ/kg DM) (**Table 1**). These wide ranges of nutritive value suggest that there is an opportunity to increase its quality to a considerable extent when managed properly. Therefore, it is necessary to understand different management factors that contribute to high or low yield and their impact on the nutritive value of this grass. Many factors such as inputs (nitrogen and water), variety, harvest management, and maturity affect the yield and nutritive value of grasses.

### 3.1 Nitrogen fertiliser

Napier, being a C_4_ grass, requires a high amount of fertiliser to achieve high yields. It requires 600 kg N/ha (Lotero et al., 1969) to 2,223 kg N/ha (Vicente-Chandler et al., 1959) to produce from 50 to 86 t DM/ha/year (**Table 2**). Goorahoo et al. (2005) recorded 78 t DM/ha/year by applying 542 kg N/ha but found that N uptake by this grass was 1,210 kg/ha. The estimated nitrogen use efficiency (NUE) of these top yielders ranged from 28 kg DM/kg N (Ferraris, 1980) to 144 kg DM/kg N (Goorahoo et al., 2005) (**Table 3**). These reports on N application and NUE suggest that the application of such a high amount of N fertiliser to Napier grass is worthy, as the highest NUE of Napier grass (Goorahoo et al., 2005; **Table 3**) was ~5 times greater than the highest NUE of Kikuyu grass (30 kg DM/kg N; Garcia et al., 2008). The mechanisms of how N fertiliser impacts N fixation and soil properties (Hu et al., 2021) including yield and forage quality (Delevatti et al., 2019) of C_4_ tropical grasses other than Napier grass have been discussed elsewhere. Overall, high yields and high NUE of Napier grass were generally achieved by using N fertiliser of 600 to 2,223 kg N/ha (depending on regions). This amount of N application is high, but Roy et al. (2016) reported that smallholder farmers in southern Bangladesh apply 1,128 kg N/ha/year to achieve 57 t DM/ha of Napier grass.

**Table 2:** Management practices of some highest- and lowest-yielding Napier grass in the literature.

References	Cultivar	Space	Irrigation	HI^¥^, days	NPK^¥^	Yield, t DM/ha/year	Plant height (cm)
Vicente-Chandler et al., 1959	NM			90	2,223 kg N/ha	85.9	
Habte et al., 2022	18,662		SWS^§^	56	Urea and diammonium phosphate (50:50) were applied at a rate of 6.2 g/plant	2.5	
			MWS^¶^			2.7	
	16,791		SWS			67.4	
			MWS			68.1	
Goorahoo et al., 2005 ^£^	Promor A		40% of evapotranspiration (ET)			52.6	
			80% of ET			74.8	
			160% of ET			78.1	
Wijitphan et al., 2009	NM	50 cm × 40 cm	Sprinkler to saturate 0–15 cm soil profile	35	Basal NPK (15-15-15) 625 kg/ha, manure basal 6.25 t/ha and then 1.56 t/ha after every 3 months; 125 kg urea/ha after each cutting	70.8	
Ferraris, 1980	Q5083				0–2,000, 150 and 800 kg NPK/ha	56	
Roy et al., 2016	NM			30–35 days in summer, 45 days in winter		314.5 t fresh (~57 t DM/ha)	
Woodard and Prine, 1993	L 79–1,002				200, 22, 83 kg NPK/ha	49	
Kabirizi et al., 2015	22 cultivars			7 harvests		17–42	
Khairani et al., 2013 ^α^	DEH				Basal manure and dolomite 440 g and 20 g/m^2^, NPK in 55 g/m^2^ in 7 splits/year	28.2	339
	DLH					13.4	160
	H					26.9	305
	PF					14.1	247
	ME					30.9	351
	WW					33.5	429
Tessema et al., 2010	ILRI 16,791	100 cm × 50 cm		60	Urea 50 kg/ha; P, 100 kg/ha	16.4	
				90		25.8	
				120		31.7	
Muyekho et al., 2015	Ouma			4 harvests	P_2_O_5_ 60 kg/ha, N 100 kg/ha as calcium ammonium nitrate	23–29	
	Bana					19–34	
	South Africa					24–33	
Wamalwa et al., 2015	25 cultivars					6.8–23.8	

^¥^HI, harvest interval; NPK, nitrogen, phosphorous, potassium; NM, not mentioned; SWS^§^, severe water stress with 10% volumetric water content applied during dry season from November to May but rainfed in other seasons; MWS^¶^, medium water stress with 20% volumetric water content applied during dry season from November to May but rainfed in other seasons; ^α^DEH, Dwarf early heading; DLH, Dwarf late heading; H, hybrid (pearl millet × Napier); PF, purple foliage; ME, Merkeron; WW, Wruk Wona; ^£^N 341–542 kg/ha, P 185 kg/ha; N and P absorbed, 1,210 and 258 kg/ha.

**Table 3:** Nutrient use efficiency of some top-yielding Napier grass.

	Yield t DM/ha	N fertiliser kg/ha	NUE^α^ kg DM/kg N	Total water (mm/ha)	WUE^β^ (t DM/^ML^
Goorahoo et al., 2005	78.1	542	144	1,160	6.7
Roy et al., 2016	57.0	1,128	51	1,506	3.8
Vicente-Chandler et al., 1959	85.9	2,223	39		
Ferraris, 1980	56.0	2,000	28		
Lotero et al., 1969	70.0	600	117		
Average	69.4	1,298.6	75.8	1,3330	5.2
SD^µ^	13.1	780.4	51.5	244.7	1.1

^α^NUE, nitrogen use efficiency; ^β^WUE, water use efficiency; ^µ^SD, standard deviation. ML, megalitre (1 ML = 100 mm).

Nitrogen fertiliser also impacts the nutritive value of Napier grass. Several authors (Sarwar et al., 1999; Zewdu et al., 2002) reported that N fertiliser increased CP content, but Zewdu et al. (2002) did not observe any effect of N fertiliser on ash, ADF, NDF, cellulose, hemicellulose, calcium (Ca), phosphorous (P), *in vitro* DM digestibility (IVDMD), or ME content.

Nitrate nitrogen (NO_3_–N) and oxalate content of Napier grass have significant impacts on animal nutrition. Napier grass, on average, contains 0.5 g/kg DM NO_3_–N (0.1–0.8 g/kg DM; **Table 1**). It appears that the NO_3_–N content of this grass is at the safe level for animals, as Burrows and Tyrl (1989) reported that the safe limit of NO_3_–N is 2.5 g/kg DM and that forages exceeding 4.5 g/kg DM are highly toxic to animals. Adams et al. (2019) also suggested that the NO_3_–N content of forages causing acute toxicity generally ranges from 2.3 to 6.8 g/kg DM. Although there is no information on the impact of N fertiliser on the NO_3_–N content of this grass, Marais et al. (1987) reported Kikuyu grass applied with high N fertiliser (ammonium nitrate, 500 kg N/ha/year) at four leaf stages contained NO_3_–N 5.9 g/kg DM in the leaves and 8.8 g/kg DM in the whole plant. However, Kikuyu grass *ad libitum* in association with grain or concentrates had no issues when offered to lactating dairy cows over a long period of time (Fariña et al., 2011; Clark et al., 2015).

Napier grass usually contains 1–39 g/kg DM total oxalate and 53–60 g/kg DM silica (**Table 1**). Oxalate is known to have a negative impact on the body condition score (BCS), Ca, and P balance of cattle (Das et al., 2010; Rahman et al., 2014b). McKenzie et al. (1988) reported that plants containing 20 g/kg DM or more soluble oxalate may cause acute toxicity in ruminants. Rahman et al. (2010) reported a high total oxalate content (32–39 g/kg DM) and soluble oxalate (25–34 g/kg DM) in this grass, although oxalates were not affected by N (150 to 600 kg/ha) or potassium (K; 150 to 600 kg/ha) fertilisers. However, they (Rahman et al., 2010) grew Napier grass (cv. dwarf-late) in pots filled with sandy soils, which is likely to be attributed to the high oxalate content in this grass. Although rumen bacteria can adapt to a high level of soluble oxalate in the diet (Allison et al., 1977), sometimes, acute toxicity occurs even in adapted ruminants in diets containing relatively low oxalate (4–24 g/kg DM) concentrations (Marais, 2001). Huque et al. (2006) reported farmers complain that feeding fresh Napier grass results in weakness and poor BCS despite increased daily milk production. Das et al. (2010) reported that this weakness and poor BCS may be caused by the drainage of Ca in the form of calcium oxalate through the urine and faeces at a high rate (25 g/day). They reported that oxalate content reduces Ca and P balance in bulls, increases urinary excretion, and reduces water content in faeces. Rahman et al. (2014b) suggested supplementation of Ca source to optimise Ca balance and to improve the BCS of dairy cows. Nonetheless, they (Das et al., 2010; Rahman et al., 2014b) including Rao et al. (1993) reported positive Ca and P balance when Napier grass/silage was offered with concentrates or offered alone.

There is a paucity of data on the effect of N application (>300 kg N/ha) nutritive value, NO_3_–N, and oxalate content and their interactions with Napier grass. Further research is required on the impact of graded N fertiliser on the yield and nutritive value of this grass to optimise its yield and nutritive value.

### 3.2 Water

Napier grass requires a high amount of water for its growth. Habte et al. (2022) in a large study conducted in Ethiopia with 84 varieties reported varying yields of this grass between varieties, ranging from 2.7 (cv. 18662) to 68.1 (cv. 16791) t/ha/year (mean, 34.1 t/ha/year), but there was little yield difference within varieties due to water stress. For example, the yield of cv. 18662 was 2.7 and 2.9 t/ha and that of cv. 16791 was 67.4 and 68.1 t/ha under severe and moderate stress conditions. Severe and moderate water stress conditions were defined as 10 and 20% volumetric water content imposed in the dry season from November to May, but rainfed conditions prevailed in other seasons in both groups. Goorahoo et al. (2005) in a field experiment at Fresno, California, reported that drip irrigation at either 80% or 120% of daily measured reference evapotranspiration (ET) applied on 8-day intervals had no effect on yield. The average annual ET at Fresno is 1,264 mm (Almond Board of California, 2016), which indicates that 1,011 mm of water (80% of ET) is sufficient to optimise yield under drip irrigation. Thus, the water use efficiency (WUE) of this grass under drip irrigation is high, which is 6.7 t DM/megalitre (ML) water, even greater than the WUE of maize (4.3 t DM/ML water; Neal et al., 2011a, b). The estimated WUE of this grass was also high under farm conditions (3.8 t DM/ML water, calculated from Roy et al., 2016; mean annual rainfall, 1,422 mm, and estimated irrigation 84 mm per year; based on Meherpur Climate Bangladesh, 2023 climate data). This WUE of Napier grass on the farm is ~2–3 times greater than the WUE of Kikuyu grass (Garcia et al., 2008; 1.26–2.73 t DM/ML water). Therefore, Napier grass is a highly efficient grass in terms of its WUE both under experimental and on farm conditions.

Thus, Napier grass yields >50 t DM/ha/year with non-limiting N fertiliser (usually with >500 kg N/ha) and water (>1,100 mm) in many areas/regions of the tropics and subtropics (**Table 3**). Smallholder farmers who have access to such inputs or live in >1,100 mm rainfall zone should be able to optimise their land use by growing 50 t DM/ha/year or more compared to growing 2–10 t DM/ha/year (Bogdan, 1977) with low input in the same size of land. Thus, land-constraint smallholder farmers may increase land use efficiency, as they may grow more on the same land given that they have access to inputs.

Water also affects the quality of Napier grass varieties (Habte et al., 2022). These authors reported that Napier grass NDF, ADF, and lignin decreased but CP and ME increased under water stress conditions. The CP content was 139 and 121 g/kg DM and ME content was 8.15 and 7.65 MJ/kg DM for severe water stress and wet (rainfed plus 10%–20% water applied during the dry season from November to May) conditions, respectively. Water stress generally improves quality at the expense of yield, as water cannot serve as a carrier of nutrients required for plant growth (Islam et al., 2012).

### 3.3 Variety

Selecting the right variety can make a huge difference in increasing yield. Yield (t DM/ha) of different varieties under the same management condition ranged from 2.7 to 68.1 t (*n* = 84; Habte et al., 2022) in Ethiopia, 13 to 42 t (*n* = 80, Schneider et al., 2018) in Brazil, 17 to 42 t (*n* = 22, Kabirizi et al., 2015) in Uganda, 19 to 34 t (*n* = 3, Kabirizi et al., 2015) in Kenya, 13 to 34 t (*n* = 6, Khairani et al., 2013) in Japan, and 8 to 74 t (*n* = 18, Zhang et al., 2010) in China. This literature demonstrates that yield can simply be multiplied up to 25 times (Habte et al., 2022) by selecting the right variety. Napier grass varieties differ in plant height, leaf number, tiller number, L:S, and leaf area index (LAI; Ito et al., 1989; Zhang et al., 2010; Wangchuk et al., 2015), which impact both yield and nutritive value. Khairani et al. (2013) reported that the yield of varieties differed from 13 (cv. dwarf late heading) to 34 (cv. Wruk Wona) t DM/ha/year and also differed in plant height and LAI ranging from 160 to 429 cm and 2.5 to 8.5, respectively, and usually greater yield was associated with greater plant height. However, Gomide et al. (2015) reported that shorter varieties had a greater proportion of leaves compared to taller varieties. Zhang et al. (2010) in an experiment with 18 varieties reported that taller varieties contained a greater proportion of stem (including greater stem diameter) but fewer tillers as compared to the shorter varieties. As a result, the taller varieties (351-cm plant height) had seven times greater yield (60.6 t DM/ha/year; lines 033, 112, 121, and CK) as compared to the shorter varieties (107 cm; 8.3 t DM/ha/year; line 048), although shorter varieties contained a greater proportion of L:S (1.43:1) as compared to the taller varieties (0.35:1). Altogether, this indicates that the yield of shorter varieties is substantially lower compared to that of taller varieties, but shorter varieties are likely to be high in nutritive value as compared to the taller varieties because of their greater proportion of leaf. Land-constrained livestock farmers in the tropics likely to grow as much as possible for their livestock by compromising nutritive value unless a suitable variety that has a greater yield with a higher proportion of leaf is available.

Rengsirikul et al. (2013) conducted an experiment with eight varieties and reported wide differences in nutritive value between varieties. They reported differences in CP (62–125 g/kg DM), ash (77–116 g/kg DM), cellulose (354–473 g/kg DM), lignin (56–123 g/kg DM), and gross energy (GE, 15.0–16.4 MJ/kg DM) amongst eight varieties. Sileshi et al. (1996) also reported differences in CP (122–145 g/kg DM), ash (184–212 g/kg DM), NDF (563–642 g/kg DM), ADF (354–366 g/kg DM), lignin (48–52 g/kg DM), and IVDMD (714–748 g/kg) between three varieties (ILCA 14983, 14984, and X). Similarly, Amin et al. (2016) using four varieties (BLRI 4, Wruk Wona, hybrid Japan, and Mark Eron) reported differences in CP (104–137 g/kg DM), ash (91–116 g/kg DM), ADF (357–386 g/kg DM), total oxalate (1–8 g/kg DM), and ME (9.1–9.8 MJ/kg DM) between varieties. These data suggest that there are wide differences in yield and nutritive value between varieties and that farmers are likely to benefit by selecting the right varieties.

### 3.4 Harvest interval and maturity

The current harvest management of Napier grass is based on harvesting at different time intervals (weekly or monthly). Four to six harvests (cut and carry) per year are common (Kabirizi et al., 2015) to a maximum of 11 harvests at the experimental level (Wijitphan et al., 2009) available in the literature. All research on Napier grass in the literature showed increased yield with increased HI. Tessema et al. (2010) reported a 100% increase in yield (from 16 to 32 t DM/ha/year) when HI increased from 60 to 120 days and when plant height increased from 1 to 3 m, indicating that both yield and plant height increase with the increase in HI, and the increase in yield with increased HI is directly associated with the increased plant height. Wangchuk et al. (2015) also reported an increase in yield from 0.24 to 0.83 kg DM/plant with an increase in HI from 40 to 80 days when plant height increased from 1.5 to 2.6 m. However, this increased yield due to increased HI was associated with an 81% decrease in the proportion of leaf, which decreased from 5:1 to 0.9:1 (Wangchuk et al., 2015), indicating a substantial likely loss in quality with increased HI.

**Figure 1:**
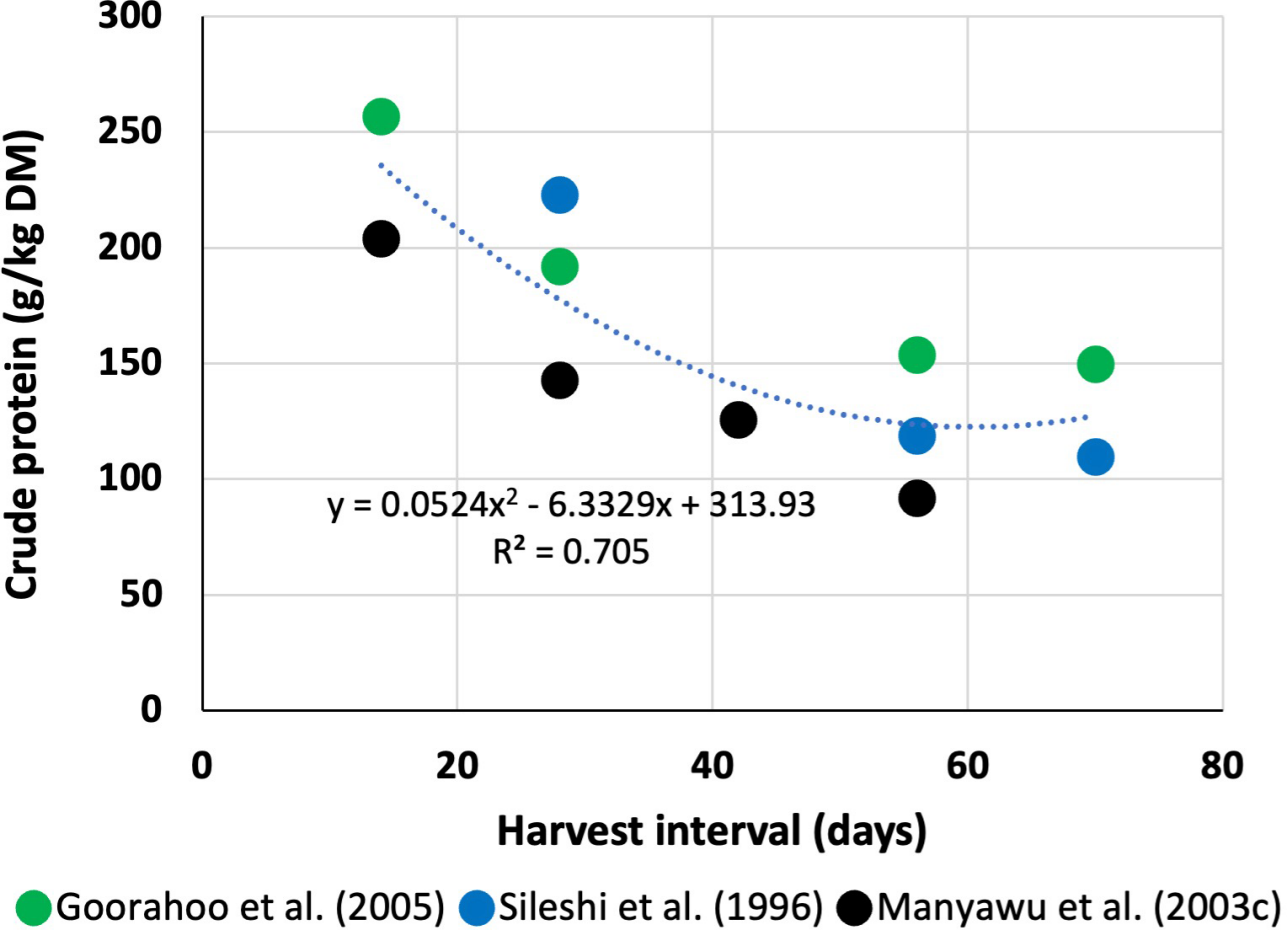
Relationship between harvest interval and crude protein content of Napier grass.

Although increased HI increases yield, researchers (Sileshi et al., 1996; Goorahoo et al., 2005) reported that increased HI (maturity) decreases both CP and energy (by increasing fibre) content of Napier grass (**Table 4**). MaChado et al. (2008) reported that organic matter digestibility (OMD) decreases from 75% to 55% with increasing maturity from 33 to 93 days. Similarly, Sarwar et al. (1999) also reported that younger Napier grass contained greater CP, dry matter digestibility (DMD) *in vivo*, and NDF digestibility (NDFD) *in situ* compared to older grasses. This reduction in nutritive value with increased maturity has an impact on animal production. Peyraud and Delagarde (2013) reported that any reductions in OMD of grass on offer will likely result in a reduction of milk yield and that a 1% reduction of OMD on grass offered involves a reduction of 1 kg milk/day. It appears that the greatest decrease (>50%) in CP (**Figure 1**) and ME and the increase in fibre occur within 56 days (from 14 to 56 days) of HI (**Table 4**). After that (from 56 to 70 days of HI), CP (**Figure 1**) and NDF decreased or increased at a slower rate (2%–7%), although ADF and lignin contents may increase 14%–27% with little or no change in the ME content of this grass. This is possible because the growth threshold diminishes at this stage (~56 days), and probably farmers, through their experiences, understand this growth threshold of Napier grass and thus harvest 6–7 times/year (i.e., 60 days of HI). Data from Tessema et al. (2010) indicate an increase in ADF (14%) and lignin (18%) and a decrease in 34% CP, which led to a decrease in digestibility *in vitro* by 11% with an increased HI from 60 to 120 days. These researchers did not find any substantial increase in NDF content or any differences in NDF and ADF digestibility *in vitro* with increased HI from 60 to 90 days, although CP decreased with increased HI. These data corroborate with others in the literature (Sileshi et al., 1996; Goorahoo et al., 2005) who found that NDF and ME do not change substantially, although ADF (0%–14%) and lignin (27%) increase and CP decreases (3%–7%) with increased HI from 56 to 70 days. This suggests that increasing HI from 60 to 120 days may change chemical composition at a slower rate compared to HI from 14 to 56 days in Napier grass. The sharp decline in CP from 14 (or 28) to 56 days of HI is likely due to the mobilisation of N [and water-soluble carbohydrate (WSC)] from the leaf for plant development (Islam et al., 2012) or regrowth (Islam et al., 2020), which ultimately increases the fibre and reduces ME content. These results suggest that a better harvest management strategy is required to optimise the nutritive value of this grass for milk or meat production.

**Table 4:** Impact of harvest interval on nutritive value of Napier grass.

	HI (days)	CP (g/kg DM)	NDF (g/kg DM)	ADF (g/kg DM)	Lignin (g/kg DM)	IVDMD (g/kg)	ME (MJ/kg DM)
Goorahoo et al. (2005)	14	257	490	300			
	28	192	490	338			
	56	154	538	367			
	70	150	546	366			
Sileshi et al. (1996)	28	223	527	290	43	803	11.7
	56	119	630	343	50	727	10.4
	70	110	597	390	63	747	10.7
Manyawu et al. (2003c)	14	204	704	360		728	10.4
	56	92	785	398		636	8.8
%increase or decrease							
Goorahoo et al. (2005)	14–56	−40	10	22			
	28–56	−20	10	9			
	56–70	−3	2	0			
Sileshi et al. (1996)	28–56	−47	20	18	15	−11	−11
	56–70	−7	−5	14	27	3	3
Manyawu et al. (2003c)	14–56	−55	12	11		−13	−13

HI, harvest interval; CP, crude protein; NDF, neutral detergent fibre; ADF, acid detergent fibre; IVDMD, in vitro dry matter digestibility; ME, metabolisable energy.

Soluble oxalate content in Napier grass is usually reduced with increased HI, affected by location, and generally, the leaf contains a greater amount of oxalate than the stem (**Table 5**; Pathmasiri et al., 2014). Nitrate–N content in young (0.7 g/kg DM) Napier grass (age not defined) was greater compared to that in mature (0.5 g/kg DM) Napier grass and was greater in the stem compared to the leaf (Sidhu et al., 2011), but these values were within the safe range for animals (Adams et al., 2019). However, Pathmasiri et al. (2014) reported high NO_3_–N content in Napier grass particularly in the stem (15–24 g/kg DM) at 14 days of HI, although its leaf contains ~3 times lower NO_3_–N (6–7 g/kg DM) than the stem (**Table 5**). These levels of NO_3_–N in Napier grass at 14 days of HI (Pathmasiri et al., 2014) are above its recommended level in forages (2.3–6.8 g/kg DM), causing acute toxicity in animals. However, Pathmasiri et al. (2014) found that NO_3_–N content reduces dramatically at 28 days of HI and falls far below the safe level, particularly in the leaf fraction (0.3 g/kg DM). This suggests that Napier grass to be offered before 28 days of HI should be subjected to careful monitoring of NO_3_–N.

**Table 5:** Impact of harvest interval and site of growth on nitrate–N and soluble oxalate in Napier grass plant fractions.

	Plant fractions	Site	Harvest interval		
14	28	42
Nitrate–N (%DM)	Stem	1	23.8	4.0	0.7
	Leaf	1	7.2	0.3	0.1
	Stem	2	14.9	0.6	0.2
	Leaf	2	5.6	0.3	0.1
Soluble oxalate (%DM)	Stem	1	13.4	25.8	12.0
	Leaf	1	20.2	19.8	16.8
	Stem	2	15.0	13.2	13.1
	Leaf	2	20.8	18.1	17.1

Pathmasiri et al. (2014).

However, harvesting based on fixed weekly (or daily) intervals (i.e., HI) may not be a good option due to differences in the seasonal influence of growth on Napier grass, as growth is slow in winter and high in summer. Similarly, harvesting based on plant height may also be misleading, as height is subject to change due to differences in input, management, and environmental factors. Therefore, a management strategy regarding the timing of harvest for Napier grass is required, similar to that for Kikuyu (Fulkerson and Donaghy, 2001; Garcia et al., 2014) and perennial ryegrass (Fulkerson et al., 1998) in order to maintain both yield and quality of this grass. Fulkerson and Donaghy (2001) developed the timing of harvest/grazing of grasses based on the number of leaves and reported that an ideal timing of harvesting Kikuyu and perennial ryegrass is 4.5 and 3 leaf stages, respectively, to maintain their yield and quality for animal production purposes. Fariña et al. (2011) reported that HI (or grazing interval) at these leaf stages were 26, 42, 21, and 18 days for autumn, winter, spring, and summer, respectively, for Kikuyu grass. Based on this leaf stage principle, Fariña et al. (2011) recorded 21–24 t DM/ha/year from Kikuyu grass containing 220–240 g CP/kg DM and 9.0–10.7 MJ ME/kg DM. Therefore, investigation on the impact of leaf stage-based frequent cut and carry on regrowth on Napier grass is essential to maintain both yield and quality of this grass. In addition, information on the impact of defoliation height (plant height at harvest) and severity (height from ground level at which plants are cut) of this grass is essential, as they affect subsequent regrowth (Islam et al., 2020). Moreover, research to quantify how much trade-off between leaf and stem (or yield and quality) is also required, as there is no information on this issue for Napier grass. Once this information is available, there will be opportunities to increase the yield of this grass under better management for the smallholder farmers of the tropics and subtropics provided that inputs and conditions are adequate. Therefore, it is necessary to develop a BMP for Napier grass so that the land-constrained smallholder farmers in the vast tropics and subtropics can grow more in their small patch of land for animal production and to increase milk and meat.

### 3.5 Plant density

Wijitphan et al. (2009) demonstrated an increased planting density from 50 cm × 100 cm to 40 cm × 50 cm increased yield substantially from 56 to 71 t DM/ha/year when harvested at 35 days of HI. They reported that greater plant density ensured greater tiller number per unit area and possibly greater radiation use, which helped to increase yield. Such increased frequency of harvesting (i.e., 10–11 times/year when harvested at 35 days of HI) has the potential not only to supply year-round grass for farmers but also to increase quality compared to the current four to six harvests. In addition, because of harvesting at 35 days of HI, they (Wijitphan et al., 2009) also achieved a relatively high CP (135 g/kg DM) and ME (calculated, 10.8 MJ/kg DM; IVDMD 75.5%) in both density treatments. It is likely that increasing plant density compensates for yield (which is currently obtained by plant height or HI) and that frequent harvesting compensates for quality (particularly CP and energy). This suggests that a management strategy of increased plant density and HI can increase both yield and quality.

## 4 Potential in saline and temperate zone

### 4.1 Salinity

Salinity is one of the leading threats in the agricultural system. Irrigated lands, which produce one-third of the world’s food, are particularly prone to salinity, and 20%–50% of the world’s irrigation schemes are salt affected (Munns, 2011). As such, over 6% of the land in the world is salt affected, and this is increasing through agricultural practices (Bromham and Bennet, 2014). However, grasses particularly, C_4_ grasses, are more salt tolerant compared to cereal crops or C_3_ grasses. Bromham and Bennet (2014) in an extensive experiment reported that C_4_ grasses are more salt tolerant compared to C_3_ grasses. They reported that greater water use efficiency of C_4_ photosynthesis lowers the flux of water and salts through the plant per growth unit and reduces the ionic stress through decreasing transpiration rates, which can reduce the amount of salt in C_4_ grasses. Napier grass as a high water use-efficient C_4_ grass grows well in saline areas (Rahman and Talukder, 2015). Rahman and Talukder (2015) reported a maximum of 45.5 t DM/ha/year (204 t fresh/ha, 22.3% DM) when grown between 5 and 10 deci-Siemens/m saline areas of coastal Bangladesh and harvested at 40–45 days of HI. As the WUE of Napier grass is greater than many C_4_ grasses (Section 3.2), there is great potential for this grass in the coastal areas for livestock production.

### 4.2 Temperate region

High yield from this grass can be achieved in temperate regions through strategic management. Ito and Inaga (1988) compared the yield of Napier grass between temperate Tokyo and tropical Miyazaki in Japan and reported that 39 t DM/ha/year can be achieved in Tokyo during summer months compared to 52 t DM/ha/year in Miyazaki. They reported lower temperature and radiation and shorter day lengths in Tokyo in winter compared to Miyazaki, but temperatures were similar between the sites in summer. Despite similar temperatures between sites in summer, plant growth rate owing to their increased LAI was greater in summer in Tokyo than in Miyazaki (Ito et al., 1989).

However, Napier grass is winter dormant and sensitive to frost, so little growth occurs at <15°C, and its growth ceases at 10°C (Duke, 1983). Therefore, there is a shortage of grass for the smallholder farmers in dry winter seasons particularly during September–October months, but excessive growth occurs in wet and rainy seasons (Njarui et al., 2010) in the tropics. We observed farmers in Bangladesh and found that taller varieties grow better in winter than shorter varieties, as shorter varieties start flowering at shorter heights in winter. Thus, farmers do not obtain sufficient grass from shorter varieties for their livestock in dry winter months when there is a shortage of grass. Research is required to select breeds or varieties that perform well at low temperatures, contain a greater proportion of leaf for quality but do not compromise yield (or less compromising), and make hay or silage from this grass at the time of excess growth.

## 5 Best management practice

This review identified two simple best management practices that have the potential to minimise the trade-off between yield and nutritive value for Napier grass. These are increasing plant density and harvesting frequency. With these two simple management practices, it was possible to achieve 71 t DM/ha with the potential to supply year-round forage (10–11 harvests/year; Wijitphan et al., 2009). In addition to yield, Napier grass under these management conditions contained CP 135 g/kg DM and 10.8 ME MJ/kg DM compared to 70–80 g/kg DM CP and <8 MJ/kg DM ME obtained under traditional management practices. Fariña et al. (2011), using a C_4_ grass, Kikuyu (*Pennisetum* spp.), reported that when grass and forages contained 205 g CP/kg DM and 10.2 MJ ME/kg DM and yielded 26 t DM/ha, Holstein cows were able to produce 27,835 L milk/ha. Napier grass through increased plant density and harvesting frequency maintains greater yield and quality similar to that of Kikuyu grass required for high milk yield. Therefore, more research is required to investigate Napier grass yield and quality using various inputs, varieties, and management in association with plant density and harvesting frequency.

## 6 Knowledge gaps

The following knowledge gaps were identified:

### 6.1 Inputs

There is no information on the impact of N fertiliser (>300 kg/ha) and water on major nutrients such as CP, energy, fibre, and critical nutrients such as nitrate–N, oxalate, and minerals, e.g., sodium, calcium, and phosphorous of this grass.

### 6.2 Variety

Varieties differ widely in yield, nutritive value, plant height, leaf-to-stem ratio, and nutritive value. There is no information on what characteristics should be considered to obtain both yield and quality and which varieties can overcome seasonal growth limitations, particularly in winter to ensure a year-round supply of quality forages.

### 6.3 Harvest interval and yield and nutritive value trade-off

Smallholder farmers obtain high yields from Napier grass through increased harvest interval and at the expense of high maturity under current management, which usually limits quality. A compromise between yield and nutritive value is required to obtain high nutritive value grass to support the production of different classes of animals. However, there is little or no information on management strategies on how to increase both yield and nutritive value together of this grass by identifying the ideal time of harvesting such as leaf stage, frequency of harvest, defoliation height and severity, nitrogen, or soluble carbohydrate in the stubble for regrowth.

### 6.4 Plant density management

A recent experimental plot work reported that high yield and relatively high quality of Napier grass can be obtained by increasing plant density and harvest frequency. More research is required both on the station and on the farm to optimise both yield and quality for different classes of animals.

### 6.5 Potential in saline and temperate zones

Limited evidence shows that Napier grass can be grown with relatively high yield in moderate saline areas and temperate areas during summer. More research is required in these areas.

Little or no attention has been paid to improving Napier grass’s nutritive value or to simultaneously improve yield and nutritive value. With this focus, the yield of Napier grass has increased through time at the expense of quality. Consequently, Napier grass has been portrayed as poor-quality grass, and alongside this is the inability of this grass to maintain milk or meat production. However, research on C_4_ grass conducted in Australia and elsewhere showed that both yield and quality of C_4_ grass can be improved through strategic management. Through simple changes in management such as increasing plant density and harvesting frequency (Wijitphan et al., 2009), we propose a new best management practice for Napier grass that has the potential to increase both yield and quality. This new management focused on both quality and yield has the potential to increase both milk and meat production substantially across the vast tropical and subtropical countries around the world.

## 7 Conclusions

Our review identified that Napier grass is abundant and widely popular amongst smallholder farmers in the tropics and subtropics mainly for its high biomass yield, but its quality is poor under current management, which cannot support milk or meat production of different classes of animals. Its nutritive value for animal production has been overlooked because of the complex trade-off between yield and quality. There is a lack of information on management strategies on how to increase both the yield and nutritive value of this grass. Thus, a better management strategy is required to obtain both high yield and nutritive value. All the evidence gathered in this review indicates that Napier grass’s yield and nutritive value may be improved by two simple management: increasing plant density and harvesting frequency. However, there is only one study that reported full season data on this management strategy of increased plant density and that harvesting at 35 days of harvest interval that provides 71 t DM/ha with 135 g/kg DM CP and 10.8 MJ ME/kg DM may be tested for milk and meat production. Therefore, more research on this strategy of increased plant density and harvest interval is required as to whether CP content can be increased to 170-180 g/kg DM required for lactating animals. Thus, research on the development of the “Best Management System” of Napier grass is required to optimise its yield and quality in order to optimise smallholder animal production in the tropics and subtropics, which may play a significant role in the food security of these vast areas in the world. Emphasis on developing management guidelines should be given on maximising/optimising yield and nutritive value without compromising each other. This is important, as it is hard for the land-constrained smallholder farmers to sacrifice yield. If a compromise is required, it needs to be quantified to obtain quality grass to increase milk and meat yield.

## Author contributions

MI: Conceptualization, Data curation, Formal Analysis, Funding acquisition, Investigation, Methodology, Project administration, Resources, Validation, Visualization, Writing – original draft. SG: Conceptualization, Funding acquisition, Investigation, Methodology, Project administration, Resources, Supervision, Validation, Writing – review & editing. NS: Writing – review & editing. MI: Writing – review & editing. CC: Conceptualization, Data curation, Formal Analysis, Funding acquisition, Investigation, Methodology, Project administration, Resources, Software, Supervision, Validation, Visualization, Writing – review & editing.
